# Flexural Properties and Microstructure Mechanisms of Renewable Coir-Fiber-Reinforced Magnesium Phosphate Cement-Based Composite Considering Curing Ages

**DOI:** 10.3390/polym12112556

**Published:** 2020-10-31

**Authors:** Liwen Zhang, Zuqian Jiang, Wenhua Zhang, Sixue Peng, Pengfei Chen

**Affiliations:** 1 Department of Civil Engineering, Guangzhou University, Guangzhou 510006, China; lwzhang@gzhu.edu.cn (L.Z.); zuqianjiang1996@163.com (Z.J.); sxpeng02@163.com (S.P.); s1908173850@163.com (P.C.); 2 Department of Civil Engineering, Nanjing Forestry University, Nanjing 210000, China

**Keywords:** magnesium phosphate cement, flexural strength, coir fiber, three-point bending test

## Abstract

As a renewable natural plant fiber, Coir fiber (CF) can be used to as an alternative to improve poor toughness and crack resistance of magnesium phosphate cement (MPC), replacing such artificial fibers as steel fiber and glass fiber and thereby reducing huge energy consumptions and large costs in artificial fibers’ production and waste treatment. Aiming at examining the effects of CF volume concentrations on MPC flexural properties, this study employed a typical three-point bending test, including thirty cuboid specimens, to investigate the flexural strength, load-deflection behavior, and flexural toughness of MPC with different CF volume concentrations from 0% to 4% at the curing age of seven days and 28 days. Results demonstrated that CF presented similar effects on MPC’s properties at two curing ages. At both curing ages, as CF grew, flexural strength increased first and then decreased; specimen stiffness, i.e., MPC elastic modulus, displayed a decreasing trend; and flexural toughness was improved continuously. Additionally, modern microtesting techniques, such as, scanning electron microscopy (SEM) and energy dispersive X-ray detection (EDX), were adopted to analyze the microstructure and compositions of CF and specimens for explaining the properties microscopically.

## 1. Introduction

Bearing obvious advantages in durability, setting time, early strength, and fire resistance [1,2,3] magnesium phosphate cement (MPC), as a new inorganic cementitious material, has been widely used in the rapid repair works in roads, bridges, and airstrips. However, it still has the same main drawbacks of poor toughness and crack resistance, as the other cement-based materials [4,5,6,7], which severely limits the application of MPC in practical repair works. Adding fibers to MPC is a typical and reliable method in improving such drawbacks through fibers’ “bridge effect” and sharing stress applied on MPC [7,8,9]. Ahmad et al. [10] found that basalt fiber (BF) can significantly improve the flexural strength of MPC. Feng et al. [5] demonstrated that MPC will appear ductile failure after its mixing with steel microfiber (SMF). Fang et al. [6,11] reported that glass fiber (GF) can not only increase MPC’s flexural strength but also improve its toughness. However, although all these common artificial fibers obviously have the ability to overcome these drawbacks, their higher price will still increase the cost of repair works. More importantly, the huge energy consumption caused by those fibers’ production and waste treatment will introduce severe environmental problems such as global warming [9].

Coir fiber (CF), a renewable natural plant fiber, obtained by soaking, crushing, cleaning, and drying coconut shells, is more environmentally harmless due to its better recyclability, biodegradability, and renewability [12,13,14], compared with those artificial fibers. Reis et al. [15] research shows that CF-reinforced concrete (CFRC) has better flexural strength and flexural toughness compared with other natural-fiber- (such as sugarcane fiber and banana fiber) reinforced concrete. Thanushan et al. [16] demonstrated that CF could greatly improve the residual strength, ductility, flexural toughness, and energy absorption of earth cement blocks. A study conducted by Li et al.’s [17] studies indicated that cementitious composites reinforced by CF have better flexural strength, higher energy absorption, and increased ductility. Sekar et al. [18] demonstrated that concrete incorporated CF has a greater flexural strength than that without fiber, and the flexural strength increased with curing age increasing. Additionally, Rajak et al. [19] hypothesized that randomly oriented coir-fiber-reinforced polypropylene composites offer higher mechanical properties than artificial-fiber-reinforced composites.

As a result, CF is also believed to have an ability to improve the flexural properties and toughness of MPC, which is a necessary understanding of CF effects on MPC before the application of this natural fiber in practice. Given that most existing studies only concentrate on common concrete with CF, a series of studies will therefore be carried out by us to clarify the mechanical performances of MPC mixed with CF, among which this study focusing on the effects of CF volume concentration on the flexural properties of MPC. A three-point bending test (TPBT) including thirty specimens was employed to investigate the flexural strength, flexural stiffness, flexural load-displacement response, and flexural toughness of MPC with CF of five-volume concentrations at the curing age of seven days and 28 days. The test was divided into two groups: five sets of specimens with CF from 0% to 4% at seven days and five sets of specimens with the same CF at 28 days.

## 2. Experimental Programs

### 2.1. Test Specimens 

**Figure 1:**
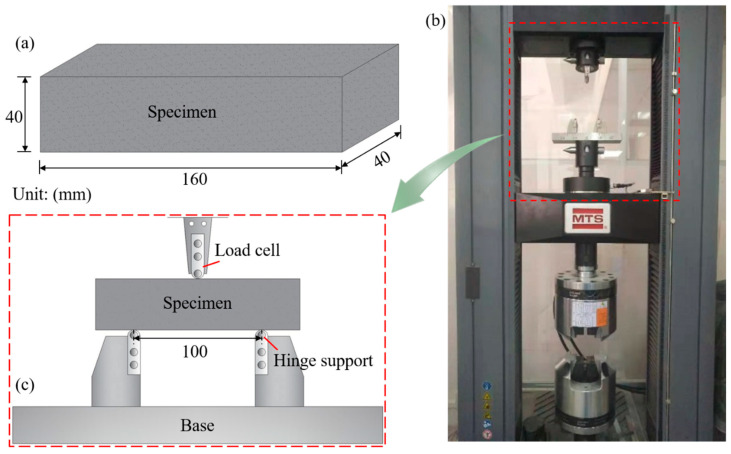
Specimen and test setup: (**a**) specimen; (**b**) testing machine; (**c**) test setup.

A total of thirty rectangular MPC blocks in ten sets were used for TPBT with the dimension of 40 mm × 160 mm × 40 mm in reference to GB/T 17671-1999 [20], as described in Figure 1a. CF volume concentration was investigated in the test respectively at the curing in a room with approximately relative humidity (RH) of 45 ± 5% at 25 ± 3 °C for seven days and 28 days. Accordingly, these ten sets of specimens were divided into two groups according to two curing ages, as summarized in Table 1. Each set consisted of three identical specimens named by the order of test type, influencing factor and its value, curing age and specimen serial number in the set. For example, FT-CF0-T7-1 means the first specimen of the set without CF subjected to the flexural test at the curing age of seven days. Basic length of CF in the test was defined as 20 mm, referring to studies related to fibers and MPC [10,21,22]. Table 1 offers more details of specimens, in which *L* and *VC* mean the length and volume concentration of CF, respectively.

**Figure 2:**
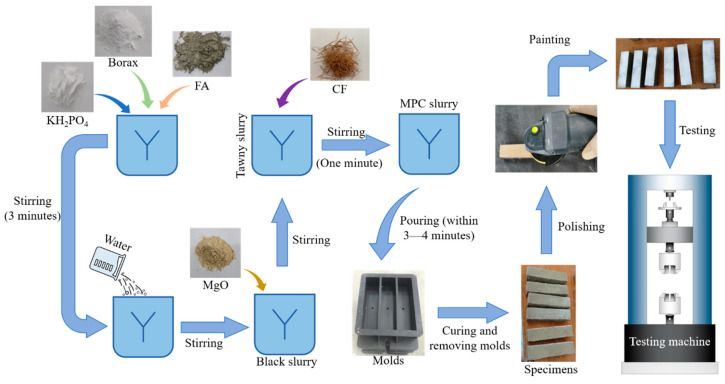
Preparation process of specimens.

In the preparation of specimens, KH_2_PO_4_, borax, and fly ash (FA) were first mixed and stirred for about three minutes. When these three materials were mixed well, water was poured into the mixture, and stirred continuously until the mixture turned to a homogeneous black slurry. Then, MgO was added and the slurry was agitated for two minutes. When the slurry turned from black to tawny, CF was dusted and then mixed well with the slurry for about one minute. At last, the mixed MPC slurry was pumped into the molds within three to four minutes. When the MPC slurry solidified completely (after approximately half to one hour), the molds were removed and specimens were cured in the time corresponding to the test requirement. Figure 2 explains the whole preparation process mentioned above.

### 2.2. Material Properties 

All MPC specimens were casted depending on a basic mix proportion used in our previous studies [23,24,25], as shown in Table 2. Dead-burned MgO (1500 °C) from a factory in Zhengyang Casting Material Company (Xinmi, China) was used in this work, with a specific surface area of 227.5 m^2^/kg, a density of 2650 kg/m^3^, and a particle size of 45 μm. Its chemical compositions are listed in Table 3. KH_2_PO_4_ and borax were produced by Jiang Hua Chemical Glass Co., Ltd. in Nanjing, China, having a purity of 99.5% and an average particle size of 350 μm. Before their blending with MgO, these two materials had been ground for six hours and dried for at least 24 h. ASTM C618 class F fly ash (FA) was employed to replace partial MgO for improving the workability of MPC mortar, its chemical compositions being shown in Table 4. 

**Figure 3:**
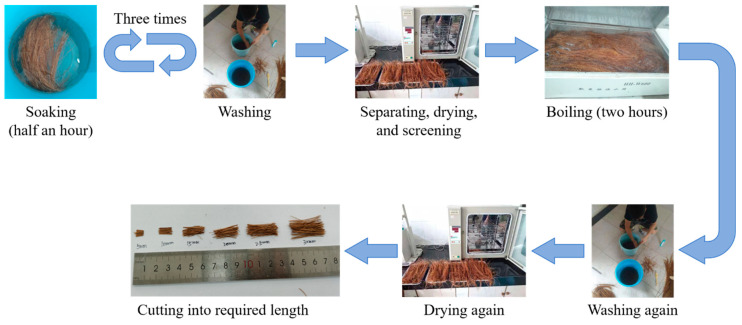
Treating process of CF.

CF applied in this study was from Sri Lanka, provided by Jia Gao Cheng (Import and Export) Trading Co., Ltd. in Jiangxi, China. Its physical properties are offered in Table 5. Before being used, CF was specially treated following the process as in Figure 3. It had been first soaked in water for thirty minutes and then washed to soften itself and remove its impurities. After repeating this soak-wash step for at least three times until the CF was cleaned and intenerated completely, it was separated with a steel comb. Then, these separated CFs were dried in a drying oven for ten to twelve hours at thirty degrees Celsius. Each CF was inspected after drying, and only the CF with a relatively uniform diameter along their axis were adopted in test to be further boiled for two hours and washed in cold water until the water ran clear. Last, these fibers were dried following the same method mentioned above and then cut to the lengths shown in Table 1.

### 2.3. Test Setup 

After specimens had been cured for the days specified in Table 1, they were employed in the test with MTS-E45.305 universal testing machine shown in Figure 1b. For ease of marking and observing their crack processes, specimens were polished and then brushed by white paint (Figure 2) before the test which was carried out in reference to ASTM-C1609 [26]. Each specimen was placed on two hinge supports with the center distance of 100 m with each support 30 mm from edge of the specimen near it, as described in Figure 1c.

After center aligning a load cell, a specimen, and a base as shown in Figure 1c, preloading was first performed at a rate of 2.0 mm/min according to the displacement of the load cell till loading force increased to 0.01 kN. Then, the loading was start at a rate of 1.0 mm/min and the load-deflection response of the specimen at its midspan was recorded by the sensor installed in the load cell. Meanwhile, a high-definition camera was set in front of the specimen to capture its fracture process. Loading would be stopped when the loading force dropped to ten percent of its peak value.

Scanning electron microscopy (SEM), energy dispersive X-ray detection (EDX), and X-ray diffraction (XRD) was carried out to investigate the effects of CF on MPC’s microstructure and hydrated products and explain the microscopic mechanism. The micromorphology studies were carried out on a system which consists of a LEO1530VP (Produced by Zeiss Co., Ltd., Jena, Germany) scanning electron microscope, equipped with a solid-state backscattered electron detector, an INCA 400 EDX (Oxford, UK) system, Pw3040 / 60 XRD powder diffractometer produced by PANalytical Co. Ltd. in Almelo, The Netherlands, was used to analyze the hydration products of each sample. The samples used for SEM and EDX were slices of 2 mm, XRD samples were grinded to 75 μm, taken from the specimen used for the flexural test. Then, the samples were put into absolute alcohol to impregnate for three days to ensure the termination of MPC hydration reaction. After the SEM samples were gold-coated and placed in SEM system devices for vacuum treatment, microscopic observation tests were carried out. The SEM image data were record by SEM-Image Processing System. XRD samples also were taken from the specimens used for flexural testing, were grinded to 75 μm, and placed in the X ray diffraction chamber to test the hydration composition at 5 to 80 degree angles.

## 3. Results and Discussion

### 3.1. General

**Figure 4:**
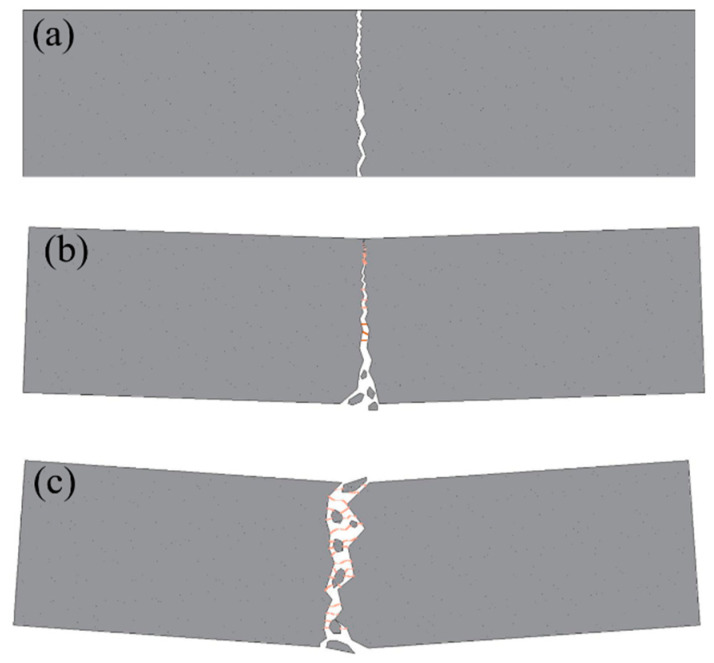
Failure types of specimens: (**a**) type I; (**b**) type II; (**c**) type III.

Based on the concentrations of mixed CF, failures of specimens could be divided into three types displayed in Figure 4a–c with respective ductility growing.

**Type I.** Specimens in this type presented a typical brittle failure. No obvious failure symptoms could be observed when the specimens were broken except for a fissure or cleft expanding throughout the specimens with a rock-fissuring sound. No pieces were peeled off from the specimens.

**Type II.** Initial signs of ductile failure appeared in this type compared with type I. A visible crack propagated to the top of the specimens at pace and expanded wide enough to form a width gap with increasing load. Soft CF-rupturing and MPC-cracking sound were heard sometimes during the test process. A few pieces fell off along the crack when the specimens failed with a small deflection. A few CF, remarked by orange lines in Figure 4, were found in the crack to connect two fracture surfaces.

**Type III.** A typical ductile failure could be observed from those specimens. A crack crawled with a significant deflection and eventually developed into a wider interstice when the specimens broke off into two parts. Fracture sounds of CF and MPC appeared more clearly and frequently in turns. Lots of unruptured CF stayed at the interstice to catch the two parts and MPC pieces peeled off.

### 3.2. Failure Modes

**Figure 5:**
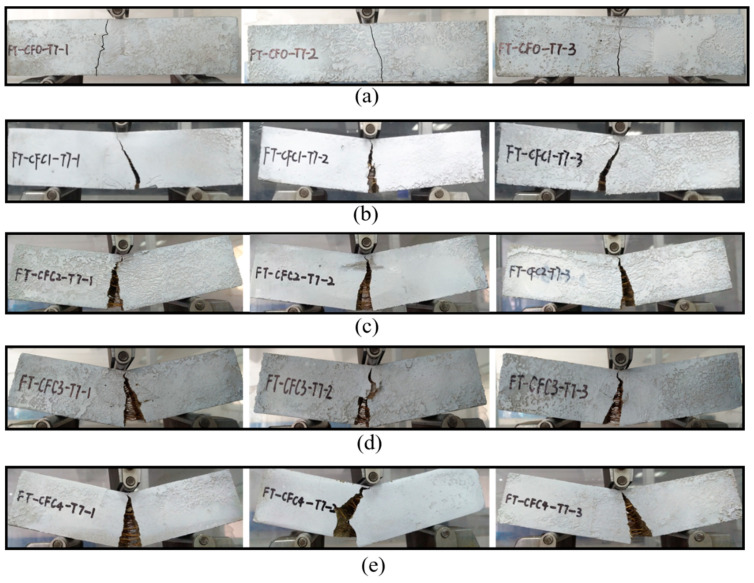
Failure modes of group T7: (**a**) CF0-T7; (**b**) CFC1-T7; (**c**) CFC2-T7; (**d**) CFC3-T7; (**e**) CFC4-T7.

Type I has been found from set CF0-T7, as described in Figure 5a, where specimens fissured on their bottom at midspan shortly after loading. In sequence, this fissure extended rapidly up and failed the specimens in seconds. Basically, no visible deflection could be observed from these failed specimens. Their load-deflection curves, as in Section 3.4, also demonstrated this failure, in which the curves declined sharply and broke at a slight deflection less than 0.4 mm.

**Figure 6:**
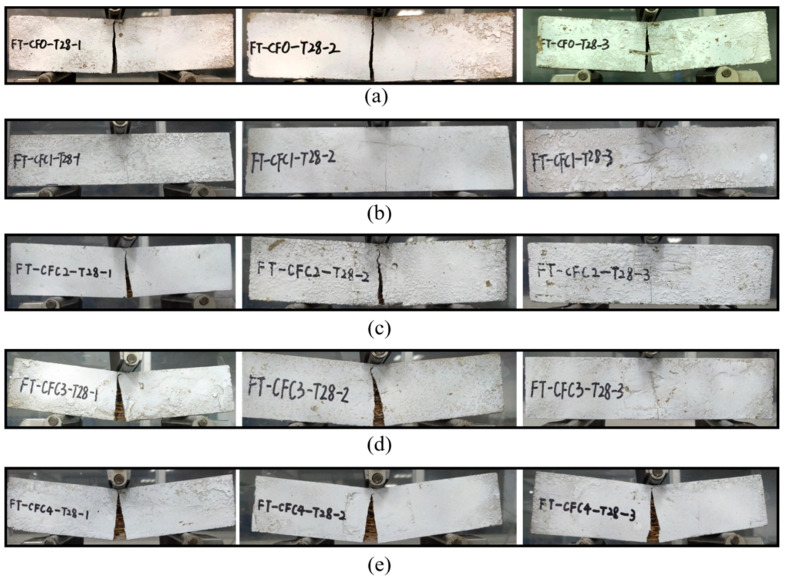
Failure modes of group T28. (**a**) CF0-T28; (**b**) CFC1-T28; (**c**) CFC2-T28; (**d**) CFC3-T28; (**e**) CFC4-T28.

Some initial signs of ductile failure, i.e., type II, could be found when a few CFs were added into the specimens. In CFC1-T7, Figure 5b, cracks occurred later than those fissures in CF0-T7 and grew relatively slowly to wider clefts. In addition, a few CF could be observed in the clefts, holding the two separated parts of CFC1-T7. With increasing CF amounts, as in Figure 6c–e, ductile failure was more obvious. For example, CFC4-T7 presented a typical ductile failure of type III. These specimens failed late, and cracks crept from their bottom to the top at the midspan. Finally, interstices with a larger width occurred in the specimens. Lots of CF stayed in these interstices, preventing specimens from breaking up.

As for specimens at 28 days, T28, they showed a similar effect of CF on failure modes with group T7. Their failure modes shifted from type I to type III with CF increasing, as in Figure 6. However, all specimens of T28 were more brittle than the specimens of T7 with the same CF concentration. Except for the specimens without CF, i.e., in set CF0-T7 and CF0-T28, each specimen mixed with CF at 28 days had a smaller deflection and narrower crack than the specimens at seven days, as demonstrated in the load-deflection curves of specimens in Section 3.4. In addition, each specimen in T28 had a sharper softening branch in its load-deflection curve than the corresponding specimen in T7 with the same CF concentration.

**Figure 7:**
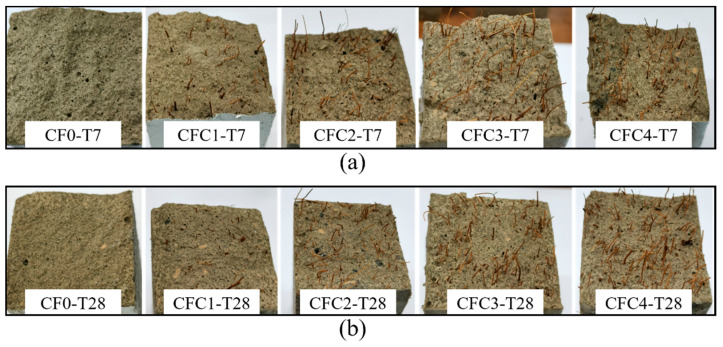
Fracture surface: (**a**) group T7; (**b**) group T28.

This increased brittleness may be attributed to the fuller hydration of MPC with increasing curing age. The fuller hydration resulted in a relatively compact structure for specimens like CF0-T28, which had a less porous fracture surface than CF0-T7, as described in Figure 7. This more compact structure was capable of improving the specimens’ loading capacity, but it also lowered their ductility. In addition, the compacter structure could result in a higher bond strength between CF and MPC. As a result, most CF were pulled apart in T28 but pulled out gradually in T7 (shown in Figure 7), leading to T28 having more brittle failures than T7.

### 3.3. Flexural Strength

**Figure 8:**
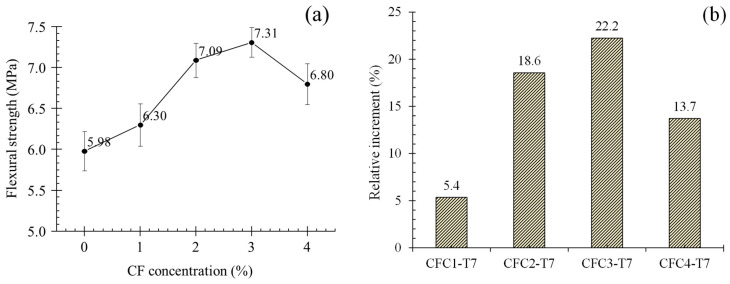
Average flexural strength and its relative increment at seven days: (**a**) average flexural strength; (**b**) relative increment.

The flexural strength of each specimen could be calculated using Equation (1) according to the Standard Test Method for Flexural Strength of Concrete (Using Simple Beam with Center-Point Loading), the code of C293, defined by American Society of Testing Materials (ASTM C293) [27].

(1) $$f_{cl}=\frac{3PL}{2bd^{2}}$$

where *f_cl_* is the flexural strength, *P* is the peak load, *L* is the distance between two supports, *b* is the specimen width, and *d* is the specimen depth. Figure 8a offers the average flexural strength (AFS) of specimens at seven days (group T7). It is obvious that appropriate concentration of CF could improve the flexural strength of MPC. As presented in Figure 8a, AFS increased from 5.98 to 7.31 MPa when CF concentration grew to 3%. Meanwhile, its relative increment to CF0-T7, as in Figure 8b, was 22.2%. However, both improvements were cut off at CF continuous increase. When CF concentration was 4%, AFS and its relative increment reduced to 6.8 MPa and 13.7%, respectively, although they were still larger than the value of CF0-T7.

That could be attributed to the “bridge effect” [28,29] of CF shown in Section 4, based on which CF connected with “K-struvite”, the main hydration products of MPC. It significantly improved the tensile properties of MPC matrix comprising K-struvite columns, which originally had less resistance to tension but performed perfectly at compression, similar to calcium silicate hydrate (CSH) exhibiting an inconsiderable tension resistance in concrete and cement mortar. Correspondingly, specimens with CF displayed larger flexural strength than specimens without CF. This is because, when CF was boiled and washed, it had a more significant bridge effect due to the more regular arrangement of lignin, holocellulose, hemicellulose contents and the clean-rough surface. As a result, the flexural strength of specimens increased obviously with growing CF concentration. However, when CF was higher than 3%, excessive CF caused larger pores in the matrix shown in Figure 7a due to the poor fluidity of MPC slurry in casting and CF agglomeration, and, meanwhile, led to an increasing viscosity, poor dispersion and porosity, which decreased in the mechanical-strength-providing K-struvite [30,31]. In consequence, the flexural strength in turn dropped, although CF concentration grew to 4%. These mechanisms would be explained in more detail with microanalysis in Section 4. 

**Figure 9:**
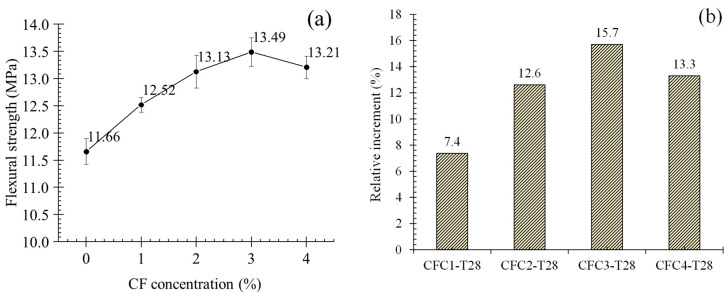
Average flexural strength and its relative increment at 28 days. (**a**) Average flexural strength; (**b**) relative increment.

For specimens at 28 days, group T28, they presented a similar trend of flexural strength with group T7, as described in Figure 9a. When CF grew to 3%, AFS increased to the maximum of 13.49 MPa, and then decreased as CF grew continuously. Additionally, relative increments for group T28 after adding CF was smaller than that of group T7. In the cases of 3% CF, AFS was raised by 15.7% in CFC3-T28, but improved by 22.2% in CFC3-T7. This may be a contribution of larger flexural strength for set CF0-T28 because of the fuller hydration.

### 3.4. Load-Deflection Curves

**Figure 10:**
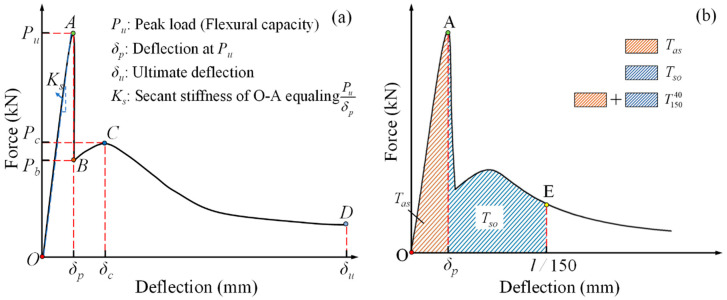
Load-deflection (L-D) curve configuration and schematic for flexural toughness calculation: (**a**) curve configuration; (**b**) flexural toughness schematic.

The load-deflection (L-D) curves of all specimens are summarized as a typical configuration consisting of four stages, as shown in Figure 10a: an approximately linear ascending stage (O–A), a sharp softening stage (A–B), a secondary nonlinear ascending stage (B–C), and a secondary gradual softening stage (C–D). For specimens without CF or with slight CF (1%), their L-D curves only displayed the first two stages, which was consistent with the obvious brittleness observed from the specimens in test, as described in Section 3. However, other specimens with CF larger than 1% improved significantly in their ductility due to the benefits from these CF and had a full L-D curve comprising all four stages. More details relative to the effect of CF on L-D curves are clarified in the following sections.

**Figure 11:**
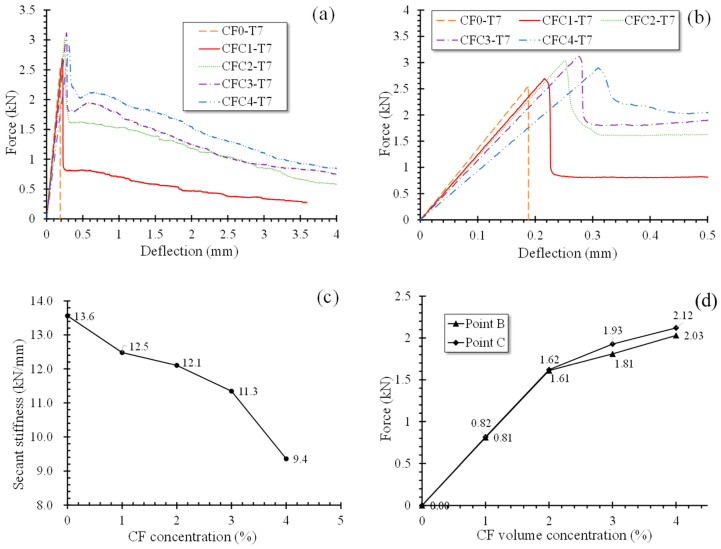
L-D curves and secant stiffness of group T7. (**a**) L-D curves; (**b**) enlarged view in O–A; (**c**) secant stiffness of O–A; (**d**) Loading force at point B and C.

Figure 11a,b provide the L-D curves of specimens at seven days and their enlarged views in stage O–A–B, respectively. All specimens had similar L-D behaviors in stage O–A and A–B except for the slopes of O–A. Loading force increased approximately linearly up to the peak load (point A) at a rapid speed, and then dropped sharply to point B. As for the slopes, i.e., the secant stiffness *K_s_* shown in Figure 11, they displayed a decreasing trend with CF growing. Figure 1c offers the relationship of secant stiffnesses and CF volume concentrations. When CF increased from 0% to 4%, *K_s_* declined from 13.6 kN·mm to 9.4 kN·mm. This dissimilar phenomenon to AFS is the result of the smaller contribution of bridge effect to raising MPC elastic modulus than to improving MPC flexural strength. Moreover, lower elastic modulus of CF will compromise the elastic modulus of MPC according to the complex material theory [32], resulting in the reduction of specimens’ elastic modulus after CF was involved, and the more significant reduction with increasing CF. Therefore, *K_s_* reduced as CF grew in the case of same-size specimens. However, lower *K_s_* implied that it may have better ductility for specimens casted by the same material. 

When curves declined to point B, however, for specimens with CF less than 1%, they would drop continuously, demonstrating the specimens was broken after a short time, but for specimens with 1% CF, they rose again from point B to point C. Moreover, this rise was more significant as CF grew, which could also be attributed to the “bridge effect” described in Section 4. Before point B, cracks did not have enough width to activate CF so that all specimens had a similar L-D behavior in stage O–A–B. However, when the load was carried on to point B, cracks propagated to a larger width, thereby causing sufficient tensile stress in CF to neutralize the flexural stress in MPC. Besides, this neutralized effect was improved by the increasing incorporation of CF. Figure 11d offers the loading forces at point B and point C, in which an obvious increase of the loading forces could be found with growing CF. For specimens with lager CF volume dosage, result showed that they had an ability to resist load sequentially and demonstrated secondarily ascending L-D curves from point B. However, after point C, CF was ruptured or pulled out due to a greater extreme tensile stress than CF tensile strength or bond capacity with MPC, leading to the curves descending again. Similarly, stage C–D descended gradually with increasing. CF These phenomena suggest that, contrary to flexural strength, CF could improve the ductility of MPC sustainably, which could also be verified by the slow specimen failure process and the frequent CF-rupturing noises observed in testing.

**Figure 12:**
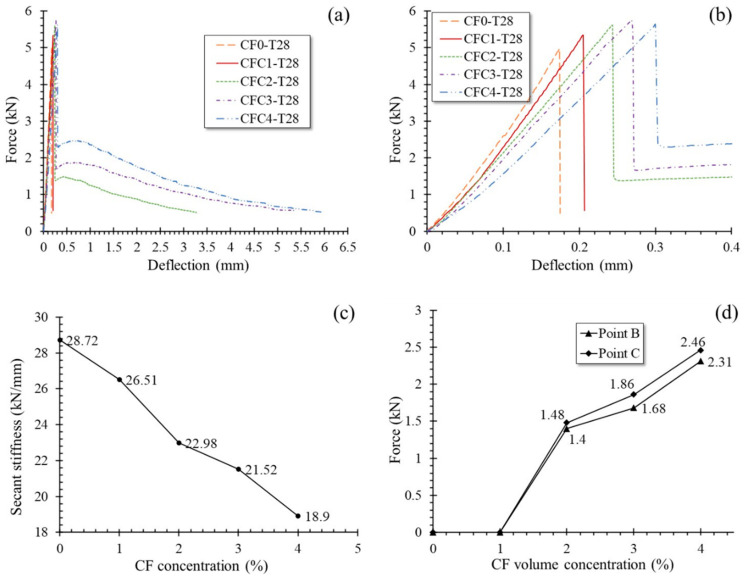
Load-deflection (L-D) curves and secant stiffness of group T28: (**a**) L-D curves; (**b**) Enlarged view in O–A; (**c**) Secant stiffness of O–A; (**d**) Loading force at point B and C.

Specimens at 28 days, group T28, had similar L-D behaviors to group T7. However, the threshold of CF concentration, which changed L-D curve configuration, increased from 1% to 2%. In the case of CF level less than 2%, i.e., sets CF0-T28 and CFC1-T28, L-D curves ascended rapidly to the peak load point like a straight line and then dropped sharply along stage A–B till the specimens failed. When CF was further added in proportions larger than or equal to 2%, L-D curves grew again from point B to point C and then declined gradually to point D where the specimen was broken. These could be clearly observed from the L-D curves of group T28 and their enlarged view in stage O–A–B described in Figure 12a,b respectively. As well as for group T7, the secant stiffness of specimens in O–A for group T28 decreased with increasing CF. Meanwhile, point B and point C rose together with the length of stages B–C and C–D when more CF was mixed, demonstrating the increasing of CF’s effectiveness on both counteracting tensile stress in MPC and improving specimens’ ductility. Figure 12c,d clearly present the phenomena mentioned above.

Additionally, group T28 expressed larger secant stiffness and shorter stage B-D than group T7 with same CF volume dosage, implying that specimens cured for 28 days were of greater elastic modulus and lower ductility. As illustrated by their failure process and modes in Section 3.2, these characteristics were the beneficiary of the compact structure of MPC undergoing fuller hydration after 28 days curing, consequently improving not only the elastic modulus of MPC itself but also the bonding strength between CF and MPC. Thus, most CF was ruptured rather than pulled out, as shown in Figure 7b, to release obviously more huge energy per unit time. On that ground, T28 showed lower ductility than T7, in the case of the same CF volume dosage.

### 3.5. Flexural Toughness

The flexural toughness of specimens was obtained through calculating the area enclosed by stage O–A–E in L-D curves, *T*^40^_150_, as described in Figure 10b, according to ASTM C1609 [24], meaning the total flexural energy absorption (FE) of specimens in tests. Additionally, FE was divided into two parts for understanding the effects of CF on the energy absorption of specimens in different L-D stages. *T_as_*, the part called effective flexural energy absorption (EFE), is the energy absorption in the elastic stage O–A, marked by orange diagonals shown in Figure 10b; and the other part, *T_so_*, named residual flexural energy absorption (REF), is the energy absorption in the softening stage A–E, filled by blue diagonals.

**Figure 13:**
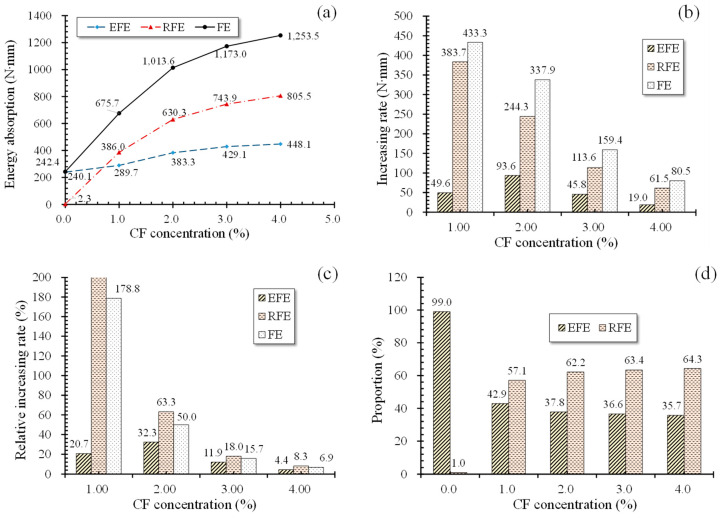
Energy absorption at seven days: (**a**) energy absorption curves; (**b**) energy absorption increasing rate; (**c**) energy absorption relative increasing rate; (**d**) proportion of flexural energy absorption (EFE) and flexural energy absorption (REF) in flexural energy absorption (FE).

Figure 13a offers the energy absorptions of specimens in different L-D stages. Obviously, FE increased with growing CF levels, suggesting a continuous improvement of the flexural toughness of specimens. However, its increasing rate exhibited a decreasing trend. Note that increasing rate was the energy absorption increment between the specimen with the present CF concentration and the specimen with the prior CF concentration. For example, the increasing rate of 1% CF was the energy absorption increment between specimens with 1% CF and 0% CF. Relative increasing rate was the ratio of the increment to energy absorptions of the specimen with the prior CF volume concentration. As shown in Figure 13b,c, FE increased by 178.8% and 433.3 N·mm when CF grew from 0% to 1% but increased by 6% and 80.5 N·mm when CF quantity rose from 3% to 4%, indicating a decline of CF’s effect on improving MPC toughness as CF increases. 

Similar to FE, EFE also grew with the increase of CF. However, its increasing rates showed a trend of rising first and then falling. Before CF was larger than 2%, the increasing rates and their relative value raised from 49.6 N·mm to 93.6 N·mm and from 20.7% to 32.3%, respectively, as CF grew from 1% to 2%. However, when CF increased to 4%, the increasing rates and the relative value dropped to 19.0 N·mm and 4.4%. This phenomenon explains CF’s significant contribution to improving microcrack resistance of MPC in the elastic behavior stage before CF exceeding 2%. Then, this contribution was reduced with CF quantity larger than 2% due to the tiny width of the microcracks. 

However, those excess CF functioned perfectly well in stages after peak load (point A). As shown in Figure 13d, the proportion of RFE in FE increased from 1.0% to 64.3% when CF grew from 0% to 4%, while the proportion of EFE decreased from 99.1% to 35.7%. Similarly, RFE showed a gradual increase like FE. Note that the data label of 1% CF was not marked in Figure 13c because it had a huge value due to the negligible RFE in the case of 0% CF. Those indicates that CF is able to improve the plastic deforming performance of MPC aside from flexural toughness, which is consistent with the failure process and modes shown in Section 3. Additionally, the increasing rates of EFE were smaller than that of FE and RFE with the same CF, demonstrating more effectiveness of CF on improving plastic deforming performance rather than on improving elastic performance.

**Figure 14:**
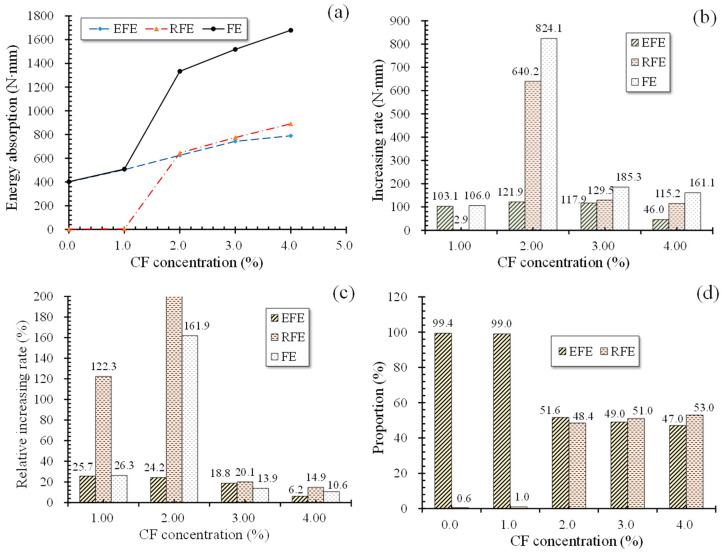
Energy absorption at 28 days. (**a**) Energy absorption curves; (**b**) energy absorption increasing rate; (**c**) energy absorption relative increasing rate; (**d**) proportion of EFE and RFE in FE.

Figure 14a offers the energy absorptions of specimens at 28 days. Generally similar to group T7, the energy absorptions of group T28 also increased with growing CF quantity, but with a slight difference that FE increased faster before CF reached 2% and then became gradually slower. Especially when CF was added from 1% to 2%, FE increased by 824.1 N·mm, a relative 161.9% increment to the case of 1% CF. That indicates an abrupt property transition of the specimen from brittleness to ductility, according to the failure modes described in Section 3.2.

Unlike FE, EFE showed a relative increasing rate decreasing with increasing CF, although its increasing rates also raised first and then decreased. Moreover, this increasing rate was raised more slightly, compared with those of T7. For example, Figure 14b displays that as CF increased from 1% to 2%, EFE grew by 121.9 N·mm, only 21.9 N·mm larger than the increment of EFE (103.1 N·mm) from 0% CF to 1% CF. This phenomenon also implies a lower effectiveness of CF on improving the crack resistance for specimens in the elastic behavior stage at 28 days than at seven days, as well as on improving the flexural strength, due to finer microcracks. Similarly, more effectiveness of excess CF was performed on the plastic deforming performance of MPC. As shown in Figure 14d, when CF grew from 1% to 4%, the proportion of RFE in FE increased from 0.6% to 53.0%, while the proportion of EFE decreased from 99.4% to 47.0%. RFE similarly showed a sharp increase when CF grew from 1% to 2% as FE, and then increased gradually with CF rising. However, although its increasing rate also slowed as more CF was incorporated when CF increased from 3% to 4%, its relatively increasing rate still stayed above 14%, as described in Figure 14c. This may have benefited from the greater bonding performance between CF and MPC due to the fuller hydration, allowing CF to play a greater role in improving the plastic deforming performance. Note that the data label of 2% CF was not marked in Figure 14c because of its huge value resulting from the negligible RFE in the case of 1% CF.

## 4. Microanalysis and Mechanisms

**Figure 15:**
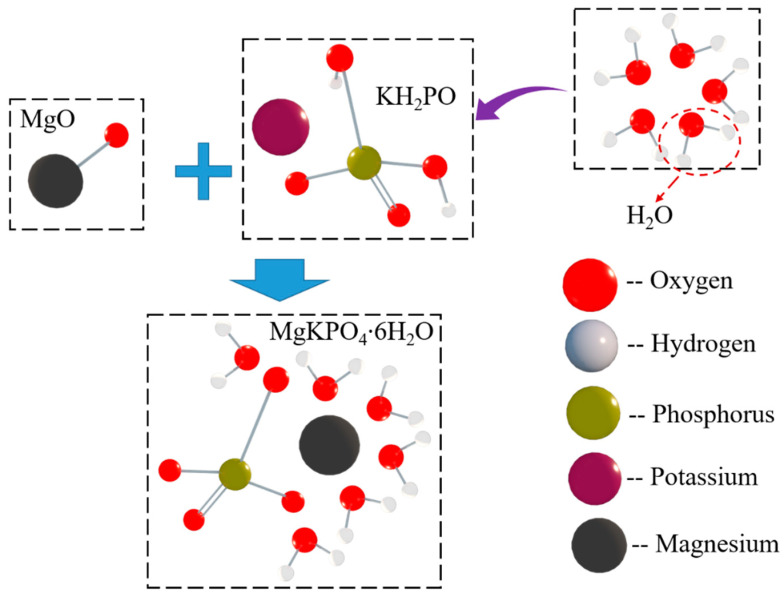
Hydration process of MPC.

To explain mechanical responses described in Section 3, a microanalysis was carried out based on modern microtesting techniques such as scanning electron microscopy (SEM), energy dispersive X-ray detection (EDX), and X-ray diffraction (XRD) to investigate the effects of CF on MPC’s microstructure and hydrated products. It is a well-known fact that, after the exciting of water, MgO undergoes a hydration together with phosphate, combining to produce a new compound named potassium phosphate magnesium (K-struvite) around unreacted MgO [33], according to the process shown in Figure 15.

**Figure 16:**
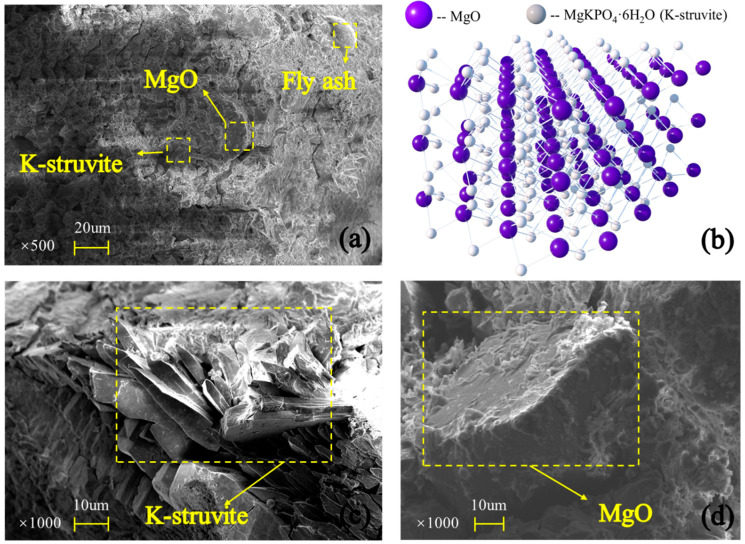
Scanning electron microscope (SEM) results: (**a**) MPC micromorphology; (**b**) schematic; (**c**) K-struvite; (**d**) MgO.

**Figure 17:**
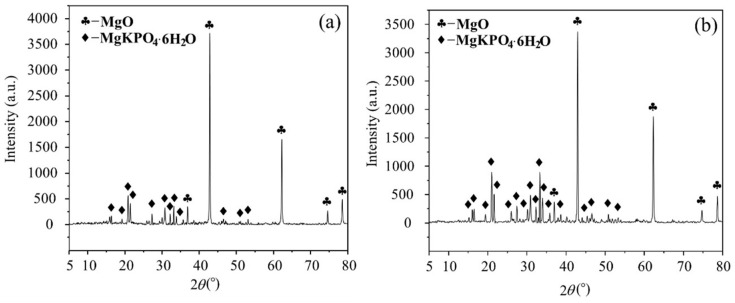
X-ray Diffraction (XRD) intensity for specimens without CF: (**a**) T7; (**b**) T28.

This compound and encased MgO form a space network structure to provide MPC mechanical strength, its SEM result and schematic offered in Figure 16a,b, respectively. However, this network composed by K-struvite and unreacted MgO (Figure 16c,d) is perfect at compression but less resistance to tension, just like calcium silicate hydrate (CSH) in concrete and cement mortar. As a result, those pure MPC specimens, CF0-T7 and CF0-T28, had a lower flexural strength, elastic modulus, and toughness. Additionally, with curing age continuing, more K-struvite was produced due to ongoing hydration. As shown in Figure 17a,b, the XRD intensity of K-struvite increased from 566 a.u. to 910 a.u. when curing age extended from seven days to 28 days, meanwhile the XRD intensity of MgO decreased from 3716 a.u. to 3371 a.u, filling the micropores between unreacted MgO and making the structure compacter as described in Section 3.2 (Figure 7). This more compact structure contributed to a larger strength, elastic modulus, and toughness of specimens with longer curing age, but reduced their ductility correspondingly, as displayed in specimens’ failure process, failure modes, and L-D behaviors in Section 3.

**Figure 18:**
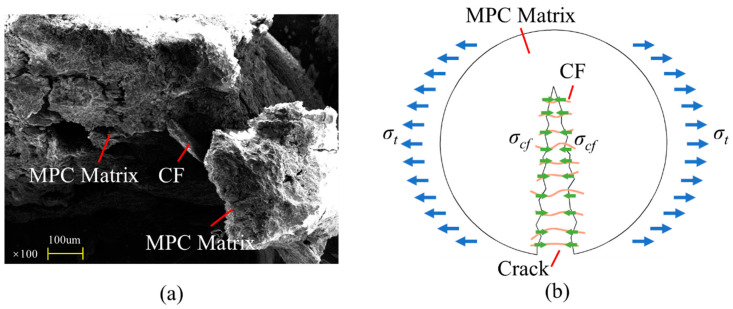
Bridge effect: (**a**) SEM for CF connection; (**b**) schematic.

In spite of that, tensile resistance would be improved after CF was added into MPC, thanks to the “bridge effect” of CF to connect MPC matrixes comprising those struvite columns. This connection shown in Figure 18a reduced tensile stress in the matrix and prevented internal microcracks from growing according to the schematic presented in Figure 18b by keeping the columns together. Besides, the effectiveness of the bridge effect mainly depended on CF’s bonding property with the matrixes and their tensile resistance.

**Figure 19:**
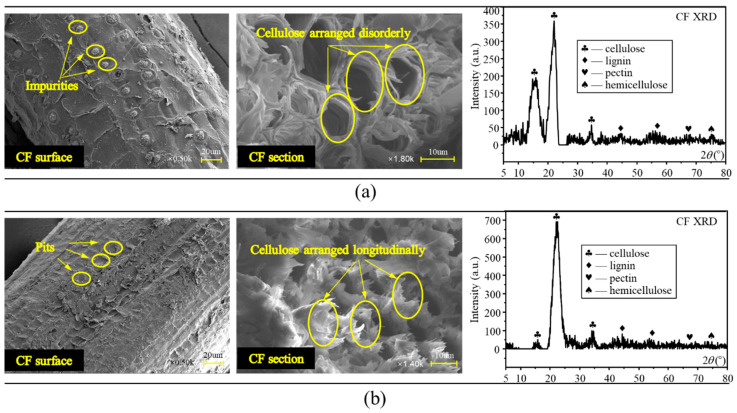
SEM and XRD for CF: (**a**) untreated CF; (**b**) treated CF.

**Figure 20:**
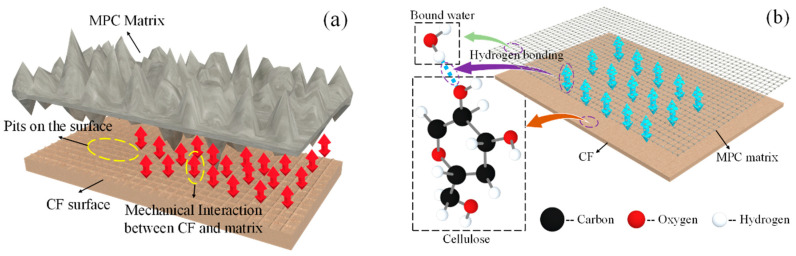
Bonding mechanism between CF and matrix: (**a**) mechanical interaction; (**b**) hydrogen bonding.

For unwashed and boiled CF, as presented in Figure 19a, some spherical particle-shaped impurities could be found on its surface, gravely weakening the bonding property. Moreover, for untreated CF, its lignin, holocellulose and hemicellulose, the main contents providing CF its mechanical strength, were in disorder arrangement, which was not conductive to CF tensile resistance, too. Therefore, CF was washed and boiled in this study to remove the impurities from its surface. As shown in Figure 19b, nothing could be observed on the CF surface except for continuous pits. In addition, lignin, holocellulose and hemi-cellulose were all arranged longitudinally, and their concentration exhibited a significant growth compared with the case of untreated CF. This rough and clean CF surface contributed to better adhesion between CF and the matrixes through improving the mechanical interaction and hydrogen bonding of the two substances shown in Figure 20a,b, and these regularly arranged CF contents were furthermore beneficial to higher CF tensile strength and toughness [34]. As a result, the effectiveness of the “bridge effect” was ensured to enhance the flexural strength, flexural toughness, and ductility when CF was incorporated. 

**Figure 21:**
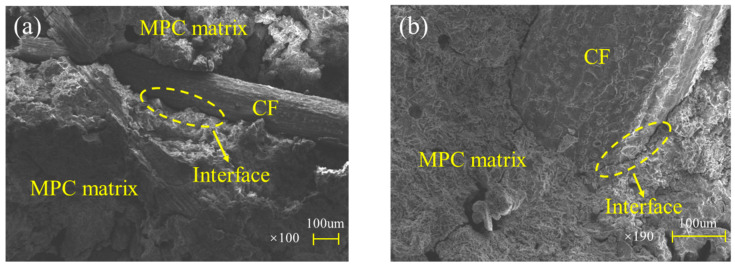
Interface between CF and MPC matrix: (**a**) T7; (**b**) T28.

A more compact structure generated from a longer curing age also brought about a better adhesion, according to the SEM results at CF-matrix interfaces shown in Figure 21a,b. In that, the matrixes attached to CF at 28 days’ curing age more tightly than that at seven days’ curing age. This better adhesion ensured CF to still work effectively even in the case of larger CF volume concentration especially in the softening stages of specimens, although it had little contribution for elastic behaviors due to negligible microcrack extension in this stage. That is why T28 presented a larger value and a higher increasing rate than T7 in EFE, RFF and FE with CF increasing, but had lower increasing rate in flexural strength, as shown in Figure 8, Figure 9, Figure 11, Figure 12, Figure 13, Figure 14.

**Figure 22:**
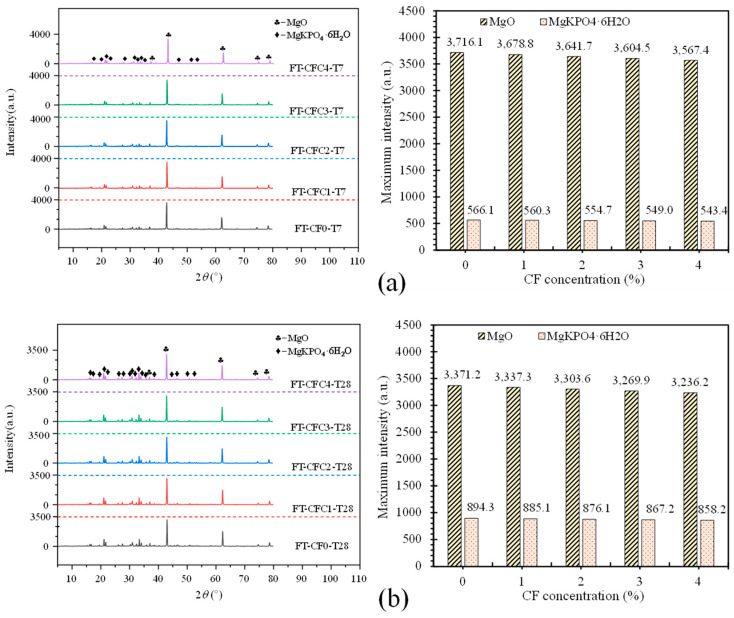
XRD results for specimens: (**a**) T7; (**b**) T28.

Although the incorporation of CF induced the improvements of MPC mechanical properties because of relying on the bridge effect, it degraded structural compactness of the matrix simultaneously. This degradation came from a significant fluidity reduction of MPC slurry in casting and severe fiber agglomeration problems due to excess CF (exceeding 3% in this study), resulting in larger pores in the matrix. In fact, the incorporation likewise reduced the amount of K-struvite but had no influence on the components of hydration products. Figure 22a,b offer XRD results of specimens at seven days and 28 days. It is clear that, taking T28 as an example, K-struvite intensity decreased from 900.43 a.u. to 840.35 a.u. with CF growing from 0% to 4%, possibly due to the contributions of two reasons: the decreasing of MPC binder (MgO and KH_2_PO) by CF replacement, and the delayed hydration reaction due to the reduction of water absorbed into CF in the reaction. These reductions of hydration products and the degradation declined flexural strength in turn when excess CF was added and lowered CF effectiveness on improving flexural toughness, as observed in Figure 8, Figure 9, Figure 11, Figure 12, Figure 13, Figure 14.

## 5. Conclusions

In this study, a three-point bending test was carried out to investigate the effects of CF on MPC’s flexural performances such as flexural strength, load-deflection behaviors, and flexural toughness. Conclusions are summarized as follows:

(1) CF presents similar effects on the flexural performances for MPC at curing ages of seven days and 28 days. However, specimens at 28 days exhibit larger flexural strength, higher stiffness, and flexural toughness than specimens at seven days.

(2) CF can improve the flexural strength but limited to its volume concentration. When volume concentration exceeds 3%, in this study, flexural strength will present a decreasing trend. Further tests are necessary to capture a more precise threshold for CF effectiveness on MPC flexural capacity.

(3) More CF incorporated contributes to improving MPC ductility, as well as reducing MPC stiffness continuously. In addition, MPC capacity displays a secondary rise after its softening first as CF increases.

(4) CF exhibits a significant contribution to improving MPC toughness. Although MPC toughness increases with more CF added, its increasing rate slows down gradually after 2% CF. In that case, MPC behaves like a ductile material. Additionally, excess CF has little help in affecting MPC’s crack resistance in its elastic stage when CF is larger than 2%.

(5) CF has less effectiveness on improving MPC elastic properties at a higher curing age but presents a significant improvement on plastic properties for them due to CF’s better bonding performance with MPC.

(6) CF reduces the amount of MPC hydration products, in spite of its negligible effect on the components of hydration products. In addition, it degrades the compactness of MPC matrix yet.

## Figures and Tables

**Table 1:** Specimen details.

Group	Set	Specimen Number	Curing Age (Day)	Coir Fiber (CF)		
				*L*^1^ (mm)	*VC*^2^ (%)	Mass (g)
T7	CF0-T7	FT-CF0-T7-1	7	0	0	0
		FT-CF0-T7-2				
		FT-CF0-T7-3				
	CFC1-T7	FT-CFC1-T7-1		20	1	3
		FT-CFC1-T7-2				
		FT-CFC1-T7-3				
	CFC2-T7	FT-CFC2-T7-1			2	6
		FT-CFC2-T7-2				
		FT-CFC2-T7-3				
	CFC3-T7	FT-CFC3-T7-1			3	9
		FT-CFC3-T7-2				
		FT-CFC3-T7-3				
	CFC4-T7	FT-CFC4-T7-1			4	12
		FT-CFC4-T7-2				
		FT-CFC4-T7-3				
T28	CF0-T28	FT-CF0-T28-1	28	0	0	0
		FT-CF0-T28-2				
		FT-CF0-T28-3				
	CFC1-T28	FT-CFC1-T28-1		20	1	3
		FT-CFC1-T28-2				
		FT-CFC1-T28-3				
	CFC2-T28	FT-CFC2-T28-1			2	6
		FT-CFC2-T28-2				
		FT-CFC2-T28-3				
	CFC3-T28	FT-CFC3-T28-1			3	9
		FT-CFC3-T28-2				
		FT-CFC3-T28-3				
	CFC4-T28	FT-CFC4-T28-1			4	12
		FT-CFC4-T28-2				
		FT-CFC4-T28-3				

^1^ L means the length of coir fiber (CF); ^2^ VC represents the volume concentration of CF.

**Table 2:** Mix proportion of magnesium phosphate cement (MPC) (kg/m^3^).

MgO	KH_2_PO_4_	Borax	Fly Ash (FA)	Water
1171.87	797.26	117.18	295.31	328.3

**Table 3:** Chemical compositions of MgO.

**Compositions**	MgO	Al_2_O_3_	Fe_2_O_3_	CaO	SiO_2_	LOI
**Mass of Concentration (%)**	96.25	0.29	1.09	1.18	1.16	0.03

**Table 4:** Chemical compositions of fly ash (FA).

**Compositions**	SiO_2_	Al_2_O_3_	Fe_2_O_3_	CaO	TiO_2_	MgO	SO_3_	LOI
**Mass of Concentration (%)**	56.74	24.58	6.55	4.87	1.86	3.3	0.8	1.3

**Table 5:** Physical properties of coir fiber (CF).

Diameter (µm)	Density (kg/m^3^)	Tensile Strength (MPa)	Elasticity Modulus (GPa)	Elongation (%)
150–350	1200	112–146	2.3–3.4	14–28
